# Analysis of gene expression in prostate cancer epithelial and interstitial stromal cells using laser capture microdissection

**DOI:** 10.1186/1471-2407-10-165

**Published:** 2010-04-28

**Authors:** Jennifer L Gregg, Kathleen E Brown, Eric M Mintz, Helen Piontkivska, Gail C Fraizer

**Affiliations:** 1 School of Biomedical Sciences, Kent State University, Kent, OH, USA; 2 Department of Biological Sciences, Kent State University, Kent OH, USA

## Abstract

**Background:**

The prostate gland represents a multifaceted system in which prostate epithelia and stroma have distinct physiological roles. To understand the interaction between stroma and glandular epithelia, it is essential to delineate the gene expression profiles of these two tissue types in prostate cancer. Most studies have compared tumor and normal samples by performing global expression analysis using a mixture of cell populations. This report presents the first study of prostate tumor tissue that examines patterns of differential expression between specific cell types using laser capture microdissection (LCM).

**Methods:**

LCM was used to isolate distinct cell-type populations and identify their gene expression differences using oligonucleotide microarrays. Ten differentially expressed genes were then analyzed in paired tumor and non-neoplastic prostate tissues by quantitative real-time PCR. Expression patterns of the transcription factors, *WT1 *and *EGR1*, were further compared in established prostate cell lines. WT1 protein expression was also examined in prostate tissue microarrays using immunohistochemistry.

**Results:**

The two-step method of laser capture and microarray analysis identified nearly 500 genes whose expression levels were significantly different in prostate epithelial *versus *stromal tissues. Several genes expressed in epithelial cells (*WT1, GATA2*, and *FGFR-3*) were more highly expressed in neoplastic than in non-neoplastic tissues; conversely several genes expressed in stromal cells (*CCL5, CXCL13, IGF-1, FGF-2*, and *IGFBP3*) were more highly expressed in non-neoplastic than in neoplastic tissues. Notably, *EGR1 *was also differentially expressed between epithelial and stromal tissues. Expression of *WT1 *and *EGR1 *in cell lines was consistent with these patterns of differential expression. Importantly, WT1 protein expression was demonstrated in tumor tissues and was absent in normal and benign tissues.

**Conclusions:**

The prostate represents a complex mix of cell types and there is a need to analyze distinct cell populations to better understand their potential interactions. In the present study, LCM and microarray analysis were used to identify novel gene expression patterns in prostate cell populations, including identification of WT1 expression in epithelial cells. The relevance of WT1 expression in prostate cancer was confirmed by analysis of tumor tissue and cell lines, suggesting a potential role for WT1 in prostate tumorigenesis.

## Background

Prostate cancer is the most common cancer in men, with over 186,000 people affected annually and a lifetime risk of 1:6 [1]. Mechanisms of prostate cancer development and progression vary and are not well understood. With age, normal prostate epithelial structure often changes, resulting in benign or malignant consequences. Benign prostatic hyperplasia (BPH) is characterized by prostate enlargement due to proliferation of epithelia; cells preserve their normal characteristics and do not progress to malignancy. Alternatively, prostate epithelia may accumulate any number of genetic changes leading to carcinogenesis. Prostatic adenocarcinoma is characterized by invasion of the underlying stroma by malignant epithelial cells (reviewed in [2].). Prostate carcinoma can be classified according to the features of malignant acini; stage T2 tumors are confined within the prostate, while advanced stage T3 tumors spread into the adjacent tissue.

The prostate gland is composed primarily of epithelial and interstitial stromal cells. Communication between these cell types is important not only for normal development, but also for prostate tumorigenesis [3]. Prostate epithelial cells are primarily luminal but include a mixture of basal and neuroendocrine cell types [4,5]. The surrounding adjacent stromal cells, which are a mixture of fibroblasts, smooth muscle, endothelial, nerve, and inflammatory cells [4,6,7], influence the growth and development of prostate cancer epithelial cells and affect androgen responsiveness [8]. Typically, studies have utilized surgically dissected samples that included mixtures of cell types [9,10]. As such...., microarray analyses comparing these "tumor" with "normal" samples are difficult to interpret, since gene expression in tumor epithelial cells was diluted by the inclusion of adjacent stromal cells in the analysis, leading to ambiguous results. Thus, a true assessment of differential gene expression in tumor tissue requires cell-specific comparisons.

The identification of distinct gene expression patterns in tumor epithelia and adjacent stroma can help elucidate cell communication pathways that are active in prostate cancer. Previous studies using laser capture microdissection (LCM) have examined differential gene expression between stromal samples, either prostate stroma relative to bladder stroma [11] or reactive tumor stroma relative to normal stroma [12]. Other studies have enriched tumor epithelial cell populations using LCM, but have made comparisons between different Gleason grades [13] or between different treatments [14]. Additional studies have utilized different tissue sources (such as formalin fixed paraffin embedded tissue [15-17] or frozen biopsies [18]) or tested different platforms (such as cDNA arrays [19]). There was also one report comparing expression in untreated prostate tumor stroma compared to tumor epithelia [20]; however the 5 microdissected tissues samples were pooled precluding statistical analysis. Thus, although several studies have addressed differences in gene expression between various epithelial or stromal populations, currently very little is known about differences between stroma and epithelia.

Given the need to identify specific gene expression patterns in both tumor epithelial and adjacent stromal cells, we chose to isolate cells of these tissue types using laser-capture microdissection (LCM). While this study analyzed differences in gene expression between microdissected tumor epithelial cells and adjacent stromal cells within the neoplastic prostate, a major focus of this study was to identify genes whose expression was enriched in stromal compared to epithelial cells. Another aim was to determine whether some of the genes previously described as "expressed in prostate cancer" were actually expressed to a greater extent in stromal tissues than in epithelial. Microdissection of specific cells within the prostate tumor and subsequent microarray analysis more accurately identified expression of major genes in prostate cancer whose expression was limited to specific cell populations. Growth factor signaling and transcription factor regulatory genes were two gene categories identified by this microarray analysis. Additionally this approach identified differential expression of the transcription factor, WT1, in prostate cancer epithelial cells and lead to subsequent characterization of its expression in cell lines and in paired non-neoplastic and tumor frozen biopsies.

## Methods

### Tissue Acquisition

All tissues were acquired and used with IRB approval from Kent State University and the appropriate institutions (see below). Frozen tissues in optimal cutting temperature media (OCT) were obtained for RNA isolation while formalin fixed paraffin embedded (FFPE) tissues were obtained for immunohistochemistry. Two types of OCT embedded tissues were obtained: 1) 5 micron sections for laser capture microscopy (LCM) and 2) OCT blocks for quantitative real-time PCR (QRT-PCR).

The serial frozen tissue sections for LCM were provided by The Ohio State University Prostate Cancer tissue Bank, part of the Human Tissue Resource Network (HTRN) in the Department of Pathology (Columbus, Ohio). The tumor samples were removed during radical prostatectomy and frozen in OCT. Tumors were categorized as intermediate grade (primarily Gleason grade 3). Two of three samples had a combined Gleason score of 6 and one had a GS 7. One of the serial sections from each tumor was stained with hematoxylin and eosin and the tumor areas marked for identification. Stromal tissue of all 3 samples appeared to contain a similar proportion of inflammatory cells.

For QRTPCR analysis twenty paired prostate tissues were provided by Dr. C. Magi-Galluzzi (Cleveland Clinic Foundation, Cleveland, OH). Tissues were obtained by radical prostatectomy, paired tumor and non-neoplastic tissues were selected from each prostate and frozen in OCT. All tumor samples were of T2 or T3 stage with combined Gleason score of 7 and were observed to have abundant epithelial tissue for RNA isolation.

Commercially available prostate tissue microarrays (TMAs) were purchased from Creative Biolabs (Fort Jefferson Station, NY). Tissue arrays consisted of cores of formalin-fixed, paraffin embedded prostatectomy cores in duplicate or triplicate from each prostate. Cores were arrayed in a rectangular fashion and were 1.0-1.5 mm in diameter and 5 μm in thickness. A total of 31 cases of carcinoma, 7 of benign hyperplasia, and 5 normal (non-neoplastic) controls were examined. Normal samples were obtained from cancer-free prostates from normal individuals. All tissues were selected and evaluated by an independent pathologist who determined Gleason grading and differentiation status. Nearly half of the cores were from high grade tumors with Gleason scores 8-10.

### Tissue Culture

Non-neoplastic RWPE-1 cells were obtained from the American Type Culture Collection (Manassas, VA) and grown in K - SFM supplemented with 0.05 mg/mL bovine pituitary extract and 5 ng/mL EGF. Hormone responsive LNCaP tumor cells were grown in RPMI-1640 media supplemented with 10% FCS and antibiotics. Hormone insensitive LNCaP - C42, PC3, and DU145 tumor cells were grown in DME - F12 media supplemented with 10% FCS and antibiotics. All cells were maintained in 5% CO_2 _at 37°C.

### Laser Capture Microdissection

For LCM, the frozen sections were stained and dehydrated using the HistoGene LCM Frozen section staining kit as per manufacturer's recommendations. Cell capture and lysis was completed within 2 hours to assure quality RNA. The epithelial and interstitial stromal cells were isolated from ten slides containing 5 micron frozen tissue sections using an LCM microscope (Arcturus Bioscience, Mt View, CA). Neoplastic areas of the slide observed to have the most abundant cells of interest were identified and marked to direct the laser capture. Stromal cells were collected from areas adjacent to glandular epithelium and included inflammatory cells. Overall, 1000 to 2000 epithelial or stromal cells were captured per cap. To verify the accuracy of capture, tissue sections and caps were examined post-capture.

### RNA Isolation and Quantification

#### Cells captured by LCM

Captured cells were lysed and RNA extracted as per manufacturer's recommendations (Arcturus Bioscience, Mt View, CA). Briefly, cells were incubated for 30 minutes at 42°C in Pico Pure extraction buffer. RNA purification columns were washed and treated with DNase (Qiagen Sciences, San Diego, CA). The RNA was eluted in Elution Buffer, and RNA quantity and quality were checked using the RNA Pico-Chip on the Bioanalyzer 2100 (Agilent Bioscience, Mt View, CA). RNA was amplified using the RiboAmp HS kit (Arcturus Bioscience, Mt View, CA).

#### Frozen Prostate Tissues

Frozen paired prostate tissues were removed from OCT media and RNA isolated using the RNEasy Mini Kit per the manufacturer's recommendations (Qiagen, San Diego, CA). Briefly, tissues were homogenized by sonication. RNA was purified by several washes in the RNEasy mini column and eluted with water. RNA quantity and quality was measured with RNA MicroChips using the Bioanalyzer 2100 per the manufacturer's recommendations (Agilent Bioscience, Mt View, CA).

#### Tissue Cultures

RWPE-1, LNCaP, LNCaP-C42, PC3, and DU145 cells were grown to confluency under standard culture conditions. Cells were rinsed twice in PBS and harvested per the manufacturer's recommendations (Qiagen, San Diego, CA). RNA quantity and quality was measured as described above.

### Labeling and Oligonucleotide Microarray Hybridization

Biotin-labeled cRNA was hybridized to Affymetrix Human Genome U133A 2.0 chips (HG_U133A 2.0) for 16-hour at 45°C. The GeneChip^® ^Operating Software (GCOS) was used to run the Fluidics Station 400 and hybridized arrays were stained with the Midi_euk2v3 labeling kit for detection. The arrays were scanned using an Affymetrix^® ^GeneChip^® ^Scanner 3000. The signal intensities were normalized by Affymetrix software to the spike-controls located on the array chip. After chip normalizations, relative intensities were used to determine whether expression is absent (A), present (P), or marginal (M). Expression patterns between arrays were compared and raw signal strength was examined to verify that hybridization was effective.

### Data Analysis

Signal intensities for each gene were generated using the Microarray Suite 5.0 algorithm by Affymetrix GCOS software 1.1. In addition to the signal intensity, each gene was determined to be present, marginal, or absent using default software settings. Overlap in gene expression between epithelial and stromal cell samples was assessed by counting the number of probe sets with all three samples showing present calls. For analysis of differential expression between epithelial and stromal cell samples, a filter requiring a present call in at least 3 of the 6 arrays was applied. This reduced the total number of probe sets to be analyzed from 22,215 to 8,739. Signal intensities for the three epithelial and three stromal arrays were further analyzed using Cyber-T software http://cybert.microarray.ics.uci.edu/ using the default settings. This software generates p-values for each gene as a test of differences between groups using a Bayesian paired t-test [21]. A list of candidate differentially expressed genes was generated using genes with a posterior probability of differential expression [22] of 0.99 or higher, which corresponded roughly to a Bayes p-value of 0.001 or less.

Functional Gene Ontology (GO) annotation of genes of interest was performed using DAVID http://david.abcc.ncifcrf.gov/[23,24] and Affymetrix databases. Gene functional classification and functional annotation clustering were performed to identify functional gene groups and ontology terms that are significantly overrepresented among genes of interest.

### Quantitative Real-Time PCR

RNA samples were reverse transcribed using QuantiTect^® ^Reverse Transcription kit and DNase treatment was performed according to manufacturer's protocol (Qiagen Sciences, San Diego, CA). For LCM captured cells, pre-amplification of cDNA was done using TaqMan^® ^PreAmp Master Mix kit. Real-time PCR was performed using the TaqMan Universal Master Mix and optimized TaqMan probe sets (Table 1). Endogenous internal controls were run with every sample plate for comparisons and each sample was assayed in triplicate. Samples were amplified using the ABI 7000 thermocycler and Ct values were measured by the ABI Prism 7000 sequence detection system (Applied Biosystems, Foster City, CA). Amplification conditions were 95°C for 10 minutes, and 40 cycles of 95°C for 15 seconds and 60°C for 1 minute. The comparative Ct method (2^-ddCt^) [25] was used to analyze gene expression differences between cell types for LCM captured cells and between tumor and non-neoplastic tissues for paired frozen prostate samples. For analysis of cell lines, gene expression in tumorigenic cell lines was compared to the non-tumorigenic cell line RWPE-1. Tests of significance were done using Dunnett's two-sided multiple comparison test.

**Table 1:** Quantitative real-time PCR primer sets obtained for expression analyses (Applied Biosystems).

Functional Class	Gene(s)	ABI Assay ID^a^
Housekeeping gene	*18S*	Hs99999901_s1
	*GAPDH*	Hs99999905_m1
Zinc finger transcription factors	*WT1*	Hs002400913_m1
	*EGR1*	Hs00152928_m1
	*GATA2*	Hs00231119_m1
Growth factor signaling	*IGF-1*	Hs00153126_m1
	*IGF1-R*	Hs00181385_m1
	*IGFBP3*	Hs00181211_m1
	*FGF-2*	Hs00266645_m1
	*FGF-R3*	Hs00179829_m1
Chemokines	*CCL5*	Hs00174575_m1
	*CXCL13*	Hs00757930_m1

a. ABI (Applied Biosystems) assay ID numbers

### Immunohistochemistry (IHC) and Scoring of TMAs

Immunohistochemical staining of the prostate TMAs was performed using standard IHC techniques. Briefly, slides were deparaffinized using a sequential method of rehydration followed by antigen retrieval in citrate solution with heating. Endogenous peroxidase activity was blocked with a 3% hydrogen peroxide solution. Slides were probed with a rabbit polyclonal anti-WT1 antibody (Epitomics, Burlingame, CA). Staining was visualized using a biotinylated goat anti-rabbit IgG secondary antibody, streptavidin horseradish peroxidase solution, and DAB (Vector Laboratories, Burlingame, CA). Slides were counterstained with hematoxylin, mounted and examined by brightfield microscopy. Staining was visualized using an Olympus IX70 microscrope at 100× total magnification. Images were taken with a Diagnostic Instruments camera and analyzed using SPOT Advanced software. Immunoreactivity assessment was based on intensity of staining in epithelial cells relative to any nonspecific stromal reactivity. Slides were scored blindly by two different individuals. Relative staining intensity was scored using a 3 point scaling system, where 0 represents the absence of staining in any epithelial cells, 1 represents weak to moderate staining, and 2 represents strong staining in at least 25% of epithelial cells.

## Results

### Microarray analysis of laser captured cells

**Figure 1:**
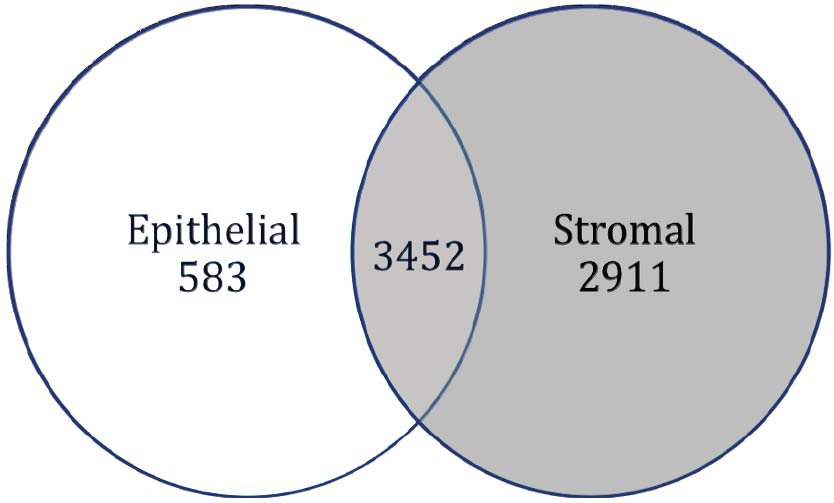
**Venn diagram of Significantly Expressed Genes**. To be considered present, the transcript had to receive a "Present" call on all three samples. 6946 of the 22215 probes on the array were identified as "present" in all 3 sample pairs (15,269 were absent or marginal) and ~50% of the identified genes (3452) were expressed in both epithelial and stromal tissue. Forty-two percent of the identified genes (2911) were more highly expressed in stromal cells (red) and only 8% (583 genes) were more highly expressed in epithelial cells (blue). The abundance of gene expression in stromal tissue is consistent with its cellular heterogeneity.

There were significant differences in gene expression between the epithelial and stromal cell samples. A *Venn *diagram was created to determine the proportion of genes expressed in common between both tissue types (Figure 1). For this figure, we utilized very stringent criteria - to be considered present, the transcript had to receive a "Present" call on all three samples from that tissue type. 6946 of the 22215 probes on the array were present in all three sample pairs for at least one of the two cell groups and half of them (3452 genes) were significantly expressed in both epithelial and stromal tissue. Reducing the stringency by allowing there to be only two present calls instead of three produces higher numbers of present probes but the trends in the data are similar. About half of the expressed transcripts were in common between two tissues types, presumably required for functions shared between these cell types. The other half represents genes likely required for cell-specific functions that were the subject of further analysis. The cellular heterogeneity of stromal tissue was consistent with the observation that ~42% of expressed transcripts (2911 genes) were elevated in stromal tissues. Conversely, only ~8% of transcripts (583 genes) were elevated in prostate cancer epithelial cells and thus comprised unique gene expression patterns. Both prostate specific genes (e.g., *PSA/Kallikrein 3 *and *Kallikrein 2*) and epithelial marker genes (e.g., *keratin 18 *and *desmoplakin*) were expressed in tumor epithelial cells. Similarly, stromal marker genes, such as *desmin *and *vinculin*, were expressed in the cells collected from adjacent stromal tissue (see additional files 1 and 2). Expression of these genes verifies the specificity of the epithelia and stroma collected.

Differential expression level comparisons of epithelial and stromal genes identified nearly 500 genes whose expression was significantly different between epithelial and stromal cells (Bayesian t-test, p < 0.001, posterior probability of differential expression > 0.99). Shown in Tables 2 and 3 are the genes with the highest probability of differential expression. Additional files 1 and 2 list 302 genes that were found to be significantly overexpressed in stromal tissue as compared to epithelial tissue, and 194 genes that were significantly overexpressed in epithelial tissue as compared to stromal tissue, respectively. To learn more about the types of gene expression differences between the two tissue types, the 496 differentially expressed genes (listed in additional files 1 and 2) were placed into functional categories using Gene Ontology (GO) Annotations.

**Table 2:** Genes Expressed significantly higher in epithelial cell samples

Probe ID	Gene Symbol	Description	Bayes p-value	Fold Change
205347_s_at	*TMSNB*	*thymosin, beta*	2.32E-08	43.9
214404_x_at	*SPDEF*	*prostate epithelium-specific Ets transcription factor (SAM pointed domain-ets factor)*	4.21E-08	132.1
214087_s_at	*MYBPC1*	*myosin-binding protein C, slow-type*	1.23E-07	75.5
202489_s_at	*FXYD3*	*FXYD domain containing ion transport regulator 3*	1.78E-07	36.6
218211_s_at	*MLPH*	*melanophilin*	1.85E-07	27.3
209706_at	*NKX3-1*	*NK3 transcription factor related, locus 1*	2.72E-07	28.1
204379_s_at	*FGFR3*	*fibroblast growth factor receptor 3*	7.91E-07	259.5
217771_at	*GOLPH2*	*golgi phosphoprotein 2*	8.28E-07	36.3
201196_s_at	*AMD1*	*adenosylmethionine decarboxylase 1*	9.01E-07	15.6
201839_s_at	*TACSTD1*	*tumor-associated calcium signal transducer 1*	1.06E-06	16.0
39248_at	*AQP3*	*aquaporin 3*	1.1E-06	19.6
205862_at	*GREB1*	*GREB1 protein*	1.11E-06	51.5
200632_s_at	*NDRG1*	*N-myc downstream regulated gene 1*	1.19E-06	14.3
218313_s_at	*GALNT7*	*UDP-N-acetyl-alpha-D-galactosamine:polypeptide N-acetylgalactosaminyltransferase 7*	1.43E-06	16.9
204583_x_at	*KLK3*	*kallikrein 3, (prostate specific antigen)*	1.73E-06	83.5
216920_s_at	*TRGV9*	*T-cell receptor (V-J-C) precursor*	2.07E-06	17.8
203196_at	*ABCC4*	*ATP-binding cassette, sub-family C (CFTR/MRP), member 4*	2.14E-06	26.1
209854_s_at	*KLK2*	*kallikrein 2, prostatic*	2.2E-06	95.7
209855_s_at	*KLK2*	*kallikrein 2, prostatic*	2.33E-06	74.0
201596_x_at	*KRT18*	*keratin 18*	2.41E-06	29.1
207430_s_at	*MSMB*	*microseminoprotein, beta-*	2.61E-06	64.8
204582_s_at	*KLK3*	*kallikrein 3, (prostate specific antigen)*	3.09E-06	79.4
200606_at	*DSP*	*desmoplakin*	3.18E-06	22.2
202241_at	*TRIB1*	*phosphoprotein regulated by mitogenic pathways*	3.5E-06	21.0
213920_at	*CUTL2*	*cut-like 2 (Drosophila)*	3.75E-06	18.6
211144_x_at	*TRGV9*	*T-cell receptor (V-J-C) precursor*	3.79E-06	19.5
201563_at	*SORD*	*sorbitol dehydrogenase*	4.92E-06	14.6
217776_at	*RDH11*	*retinol dehydrogenase 11*	5.18E-06	11.4
221577_x_at	*GDF15*	*growth differentiation factor 15*	6.02E-06	31.7
219806_s_at	*FN5*	*FN5 protein*	6.47E-06	15.4
219049_at	*ChGn*	*chondroitin beta1,4 N-acetylgalactosaminyltransferase*	6.48E-06	13.9
202023_at	*EFNA1*	*ephrin-A1*	6.71E-06	11.1
210297_s_at	*MSMB*	*microseminoprotein, beta-*	7.34E-06	45.2
209813_x_at	*TRGV9*	*T-cell receptor (V-J-C) precursor*	9.94E-06	20.6
201690_s_at	*TPD52*	*tumor protein D52*	1.06E-05	9.5

a Probe Set ID is the identification number from the Affymetrix chip.

b. Posterior probability of differential expression > 0.99).

**Table 3:** Genes Expressed significantly higher in stromal cell samples

Probe ID	Gene Symbol	Description	Bayes p-value	Fold Change
205242_at	*CXCL13*	*chemokine (C-X-C motif) ligand 13 (B-cell chemoattractant)*	2.3E-07	200.7
202274_at	*ACTG2*	*actin, gamma 2, smooth muscle, enteric*	9.8E-07	11.9
203903_s_at	*HEPH*	*hephaestin*	1.4E-06	12.9
205132_at	*ACTC*	*actin, alpha, cardiac muscle*	1.7E-06	26.1
204655_at	*CCL5*	*chemokine (C-C motif) ligand 5*	2E-06	24.8
203413_at	*NELL2*	*NEL-like 2 (chicken)*	2.1E-06	20.1
1405_i_at	*CCL5*	*chemokine (C-C motif) ligand 5*	2.3E-06	21.8
222043_at	*CLU*	*clusterin*	2.4E-06	19.5
217764_s_at	*RAB31*	*RAB31, member RAS oncogene family*	3.5E-06	13.7
202565_s_at	*SVIL*	*supervillin*	3.8E-06	11.6
212865_s_at	*COL14A1*	*collagen, type XIV, alpha 1 (undulin)*	4.3E-06	18.0
206030_at	*ASPA*	*aspartoacylase (aminoacylase 2, Canavan disease)*	5E-06	53.5
204400_at	*EFS*	*embryonal Fyn-associated substrate*	5.2E-06	8.7
204939_s_at	*PLN*	*phospholamban*	5.2E-06	14.7
205382_s_at	*DF*	*D component of complement (adipsin)*	5.7E-06	10.3
209480_at	*HLA-DQB1*	*major histocompatibility complex, class II, DQ beta 1*	5.9E-06	22.8
201058_s_at	*MYL9*	*myosin, light polypeptide 9, regulatory*	6.5E-06	14.3
202555_s_at	*MYLK*	*myosin, light polypeptide kinase*	6.6E-06	23.6
213994_s_at	*SPON1*	*spondin 1, extracellular matrix protein*	6.9E-06	16.5
209541_at	*IGF1*	*insulin-like growth factor 1*	7.4E-06	20.9
212764_at	*TCF8*	*transcription factor 8*	7.4E-06	8.6
201105_at	*LGALS1*	*lectin, galactoside-binding, soluble, 1 (galectin 1)*	8.1E-06	9.1
205743_at	*STAC*	*src homology three (SH3) and cysteine rich domain*	9.5E-06	16.5
200897_s_at	*KIAA0992*	*palladin*	1.1E-05	7.0
201438_at	*COL6A3*	*collagen, type VI, alpha 3*	1.2E-05	7.1
205549_at	*PCP4*	*Purkinje cell protein 4*	1.2E-05	6.7
209210_s_at	*PLEKHC1*	*pleckstrin homology domain containing, family C (with FERM domain) member 1*	1.2E-05	9.5
221667_s_at	*HSPB8*	*heat shock 27 kDa protein 8*	1.3E-05	17.0
205475_at	*SCRG1*	*scrapie responsive protein 1*	1.3E-05	14.8
201540_at	*FHL1*	*four and a half LIM domains 1*	1.4E-05	11.0
214044_at	*RYR2*	*ryanodine receptor 2 (cardiac)*	1.4E-05	22.0
218087_s_at	*SORBS1*	*sorbin and SH3 domain containing 1*	1.4E-05	7.1
204083_s_at	*TPM2*	*tropomyosin 2 (beta)*	1.5E-05	11.3
218332_at	*BEX1*	*brain expressed, X-linked 1*	1.6E-05	11.9
204464_s_at	*EDNRA*	*endothelin receptor type A*	1.7E-05	7.2
204069_at	*MEIS1*	*Meis1, myeloid ecotropic viral integration site 1 homolog (mouse)*	1.7E-05	11.8

a Probe Set ID is the identification number from the Affymetrix chip.

b. Posterior probability of differential expression > 0.99).

Gene functional classification clustering analysis was performed with DAVID using two subsets of genes that were upregulated (a) in epithelial cells (*n *= 194) or (b) in stromal tissue (*n *= 302). Shown in Table 4 is a summary of these classifications (Entire list of GO_BP terms is found in additional file 3). Three gene clusters with enrichment scores greater than 1 were identified among epithelial genes (encompassing 11% of all genes), namely, membrane-associated glycoproteins (including proteases) and two groups of ion transport related genes (including metal ion and ATP dependent transporters). Notably, eleven gene clusters with enrichment scores greater than 1 were identified among stromal genes, encompassing about 28% of all stromal genes. The top three clusters included about 21 unique genes (24% of 86 grouped genes) and were comprised of collagen genes and muscle and organ development genes. Other clusters were composed of structural and intracellular matrix proteins (total of 12 genes, or 14%), immune and inflammation related genes (including MHC class II and complement components) (total of 23 genes, 27%), zinc finger transcription factors (10 genes, 12%), metal ion transporters and regulators (17 genes, 20%). The greater number of clusters identified by GO analysis of genes more highly expressed in stromal cells further demonstrates the broader diversity of gene expression patterns of the stromal tissue, due to the heterogeneous cell types that encompass the stromal compartment, supporting our earlier *Venn *analysis (Figure 1). We also found that both cell types shared some functional gene categories, such as ion transport and regulation related genes.

**Table 4:** Functional classification clustering analysis: genes differentially expressed in prostate cancer epithelial and stromal cells

Gene functional classification^a^	Number of genes (%)^b^	Gene symbols	Enrichment scores range
**I. Gene clusters upregulated in epithelial cells (40 clustered genes)^c^**			

Group 1. **Membrane-associated glycoproteins** (including proteases)	26 (65%)	*ALCAM, AQP3, C1ORF115, C20ORF3, CLDN8, DPP4, FAM134A, FXYD3, GOLM1, GPR56, HPN, KLK2, KLK3, PTPRF, SLC19A1, SLC39A6, SLC7A1, SPINT2, SYNGR2, TACSTD1, TACSTD2, TM4SF1, TMED3, TMED9, TSPAN8, YIPF1*	2.57
Groups 2-3. **Ion transporters **(including metal ion and ATP dependent transporters)	14 (35%)	*ABCC4, AQP3, ATP2C1, ATP2C2, ATP6V0E2, ATP8A1, CACNA1D, FXYD3, KCNN2, KCNN4, KCNS3, SLC39A6, SLC4A4, TRPV6*	1.34 - 2.01

**II. Gene clusters upregulated in stromal cells (86 clustered genes)^c^**			

Groups 1-3. **Organ development and structural proteins** (including muscle genes)	21 (24%)	*COL14A1, COL16A1, COL17A1, COL1A2, COL3A1, COL4A1, COL4A2, COL4A3, COL4A6, COL6A3, SLK* *ACTC1, ANGPT1, BMP5, CHRDL1, COL4A2, DES, FAM48A, MYH11, MYH6, SCRG1, SERPINF1, TPM1, TPM2*	5.87 - 7.17
Groups 4-5. **Structural and extracellular matrix proteins**	12 (14%)	*CALM3 (3 loci, Entrez Gene IDs 801, 805, 808), CETN2, EFEMP1, EFEMP2, FBLN1, MATN2, NELL2, NID2, PLS3, S100A4*	4.14 - 4.87
Group 6-7, 10. **immune and inflammation related proteins**	22 (26%)	*BTN3A2, BTN3A3, C1S, C3, C7, CCR5, CDH10, CFD, CLU, CX3CR1, CXCR4, EDNRA, FZD7, HLA-DPA1, HLA-DQA2, HLA-DQB2, IL6ST, JAM3, LPHN1, MCAM, SERPING1, SGCG*	1.9 - 3.47
Group 8. **zinc finger transcription factors**	10 (12%)	*CSRP1, CSRP2, DZIP1, FHL1, LDB3, LMO3, MBNL1, MBNL2, PEG3, ZFP36L1*	2.49
Groups 9, 11. **metal ion transporters and regulators**	17 (20%)	*ARVC2, ATP1A2, C10ORF56, CHN1, FHM2, FXYD6, ITPR1, KCNAB1, KCNMA1, KIR6.1, MBLL, PDZRN4, SERCA2, SLC24A3, SP140L, STAC, TRPC4*	1.56 - 2.06

a. Classification of 496 genes performed by DAVID Gene Ontology analysis, terms based primarily on GO_BP, GO_MF,GO_CC terms

b. %of total clustered genes in parenthesis

c. 11% of upregulated epithelial and 28% of upregulated stromal genes were clustered into 3 and 11 functional clusters, respectively

Overall, we observed that many of the differentially expressed genes identified in the "Significantly Higher" lists (additional files 1 and 2) fall into two categories that are important for cell signaling: transcription factors and growth control. Of the transcription factors identified, we examined three within the zinc finger family, namely *Wilms' Tumor 1 *(*WT1*), *GATA2*, and *early growth receptor protein 1 *(*EGR1*). Of the growth control genes identified, we examined those known to be important in prostate tumorigenesis such as the chemokines *CCL5 and CXCL13 *and members of the *insulin-like growth factor (IGF) *and *fibroblast growth factor (FGF) *signaling pathways including *IGF-1, IGF-IR, IGFBP3, FGF-2*, and *FGFR-3 *[25-27]. Based on the microarray analysis, elevated expression of the zinc finger transcription factors (*WT1, EGR1, and GATA2*) and growth factor receptors (*IGF-1R *and *FGF-R3*) was observed in the epithelia, while expression of the chemokines (*CCL5 *and *CXCL13*) and growth factor ligands (*IGF-1, FGF-2, and IGFBP3*) was found in the stroma.

In order to more precisely quantify the expression of genes in the LCM-derived samples used in the oligonucleotide microarray, we analyzed the selected genes described above using quantitative real-time PCR and the 2^-ddCt ^method [28]. Due to limitations in the quantity of RNA obtained from the laser captured samples, expression of only seven of the ten genes examined was confirmed in at least two of the three samples, namely *WT1, GATA2, CCL5, CXCL13, IGF-1, IGF-1R *and *FGF-2*. Of those genes analyzed, fold difference values were at least 1.2-fold or greater relative to the paired cell type, i.e. epithelia relative to stroma, or vice versa (data not shown). Interestingly, elevated *EGR1 *expression was not confirmed by real-time analysis of epithelial cells.

### Expression in paired tumor and non-neoplastic tissues, cell lines, and tissue microarrays

**Figure 2:**
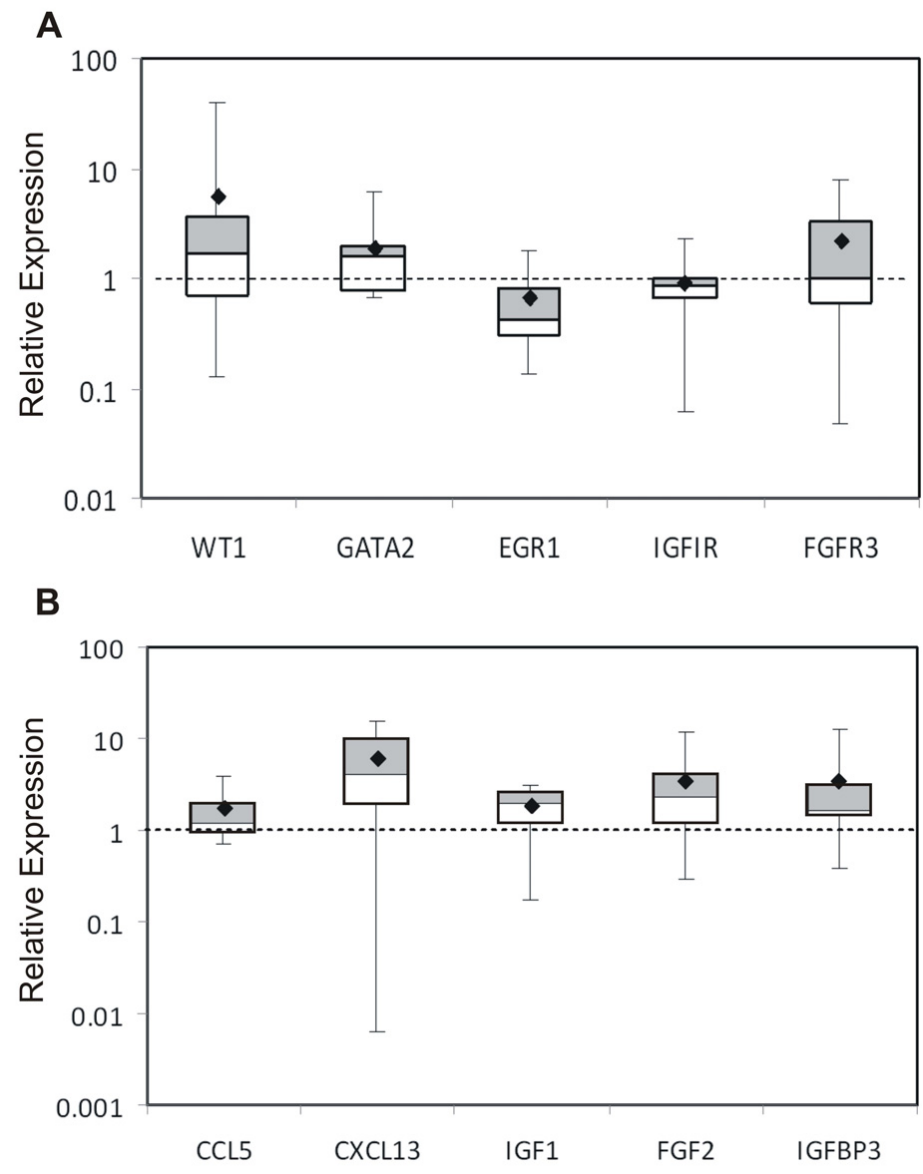
**Quantitation of differentially expressed genes in ten paired tumor and non-neoplastic samples**. **Panel A**. Relative expression of epithelial genes in tumor tissue compared to paired non-neoplastic tissue. **Panel B**. Relative expression of stromal genes in non-neoplastic tissue compared to paired tumor tissue. Data indicates fold changes in gene expression using the 2^-ddCt ^method. Values greater than 1 indicate greater expression in tumor (**A**) or non-neoplastic (**B**) tissue. The upper and lower boundaries of the boxes define the quartiles, 75% and 25%, respectively, and the black bar represents the median value. The diamond indicates the mean.

Once tissue-specific expression patterns were established, we then asked whether those genes were also expressed in normal prostate tissue. We quantified expression of the genes described above in ten paired frozen tumor and non-neoplastic prostate tissues. Expression in tumor tissue was normalized to paired non-neoplastic tissue obtained from the same prostate. The genes highly expressed in microdissected tumor epithelial cells were expected to be abundant in surgically dissected tumor tissue enriched with tumor epithelial cells. For the genes *WT1, GATA2, and FGFR3*, the expression pattern in surgically dissected tumor tissue was consistent with that in the microdissected epithelial cells (Figure 2 Panel A). In contrast, paired tissue analysis showed *IGF1R *levels in tumor tissues were similar to those in non-neoplastic samples. Another exception to the pattern of elevated expression in tumor tissues of genes identified in epithelial cells, was the significantly higher *EGR1 *expression in non-neoplastic compared to tumor tissues (p < 0.05, paired t-test). This lack of elevated *EGR1 *expression in tumor tissue was consistent with the results of the real-time PCR quantitation of expression in microdissected tissue. Overall, the expression patterns in non-neoplastic tissues from paired samples were consistent with those in stroma cells obtained from laser capture microscopy (Figure 2 Panel B). These results can be attributed to relatively fewer epithelial cells in the normal tissue samples. Thus, as a reflection of stromal cell prevalence, the stromal genes are more highly expressed in non-neoplastic tissue than in the paired tumor tissue; all mean fold difference values were 1.7-fold or greater in non-neoplastic tissues.

**Figure 3:**
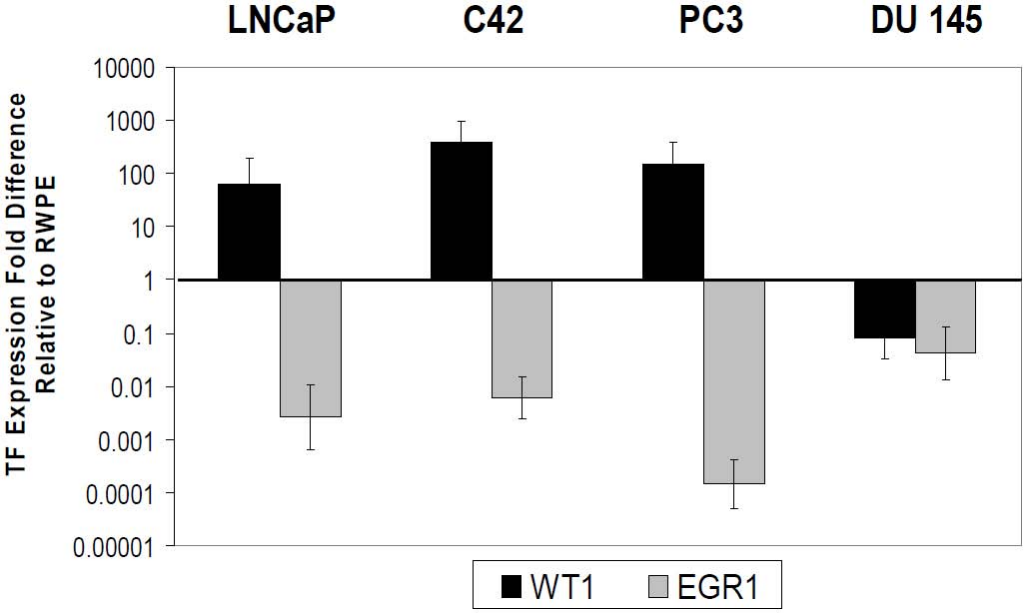
**Inverse relationship between WT1 and EGR1 in prostate cancer cell lines**. *WT1 *and *EGR1 *expression in LNCaP, C42, PC3, and DU145 prostate cancer cells was examined by QRTPCR using the 2^-ddCt ^method after normalization with 18S primers. Values shown are fold differences relative to the non-neoplastic Prostate epithelial cell line RWPE-1. All cell lines were significantly different from RWPE1 using Dunnett's Two sided multiple comparison test (p < 0.05 for *WT1 *and p < 0.001 for *EGR1*).

Additional analysis of zinc finger transcription factor expression was expanded to include another ten paired frozen tumor and non-neoplastic tissue samples. Data was analyzed by clinical stage to determine whether there was any relationship between clinical stage, especially invasiveness, and gene expression. Expression in invasive T3 stage tumors was compared to that of non-invasive stage T2 tumors. As seen in Table 5 top, in the majority of invasive stage T3 tumors, *WT1 *expression levels are higher (2.0 fold or greater) in tumor than in non-neoplastic tissues. Conversely, in the majority of localized stage T2 tumors *WT1 *expression levels are lower in tumor than non-neoplastic tissues (Table 5 bottom). Surprisingly, *EGR1 *expression was consistently lower in tumor tissues relative to non-neoplastic tissues for both stage T2 and T3 tumors. *GATA2 *expression was also reduced in tumor tissues in the majority of stage T2 samples, although in stage T3 tumors, expression was not consistent. To focus on the inverse relationship between *WT1 *and *EGR1*, we also examined five established prostate cells lines for their expression levels of each gene. Expression in the prostate tumor cell lines LNCaP, LNCaP-C42, PC3 and DU145 was normalized to expression in the non-tumorigenic cell line RWPE-1 (Figure 3). With the exception of the DU145 cells, *WT1 *and *EGR1 *expression levels were consistent with the frozen paired tissue samples examined; that is, *WT1 *expression was elevated (p < 0.05) and *EGR1 *expression reduced (p < 0.001) in tumor cell lines relative to RWPE-1 prostate epithelial cells.

**Table 5:** Quantitative real-time PCR analysis of zinc finger transcription factor expression in tumor tissue relative to non-neoplastic tissue^a^.

	Clinical stage			
	
	T3		T2	
	
Gene	Up in tumor^b,c^	No change/Down^b,d^	Up in tumor^b,c^	No change/Down^b,d^
**WT1**	7	3	3	7
**EGR-1**	0	10	2	8
**GATA-2**	4	6	2	8

a: Frozen tumor and non-neoplastic tissue obtained from 20 patients with prostate cancer (all Gleason Score 7, but clinical stage ranging from T2 to T3b). Gene expression levels determined by the ddCt method after normalization using 18s rRNA primers.

b. Number of samples with elevated expression (Up in tumor) and those with no change or reduced expression (No change/Down) in tumor relative to non-neoplastic tissue is shown for each gene and clinical stage.

c. Up in tumor: Tumor samples with elevated expression, relative to non-neoplastic tissue. Fold-change in expression values ≥ 2.0;

d. No change/Down: Tumor samples with no change or reduced expression, relative to non-neoplastic tissue. Fold-change in expression values ≥ 2.0 or no difference from non-neoplastic tissue.

**Figure 4:**
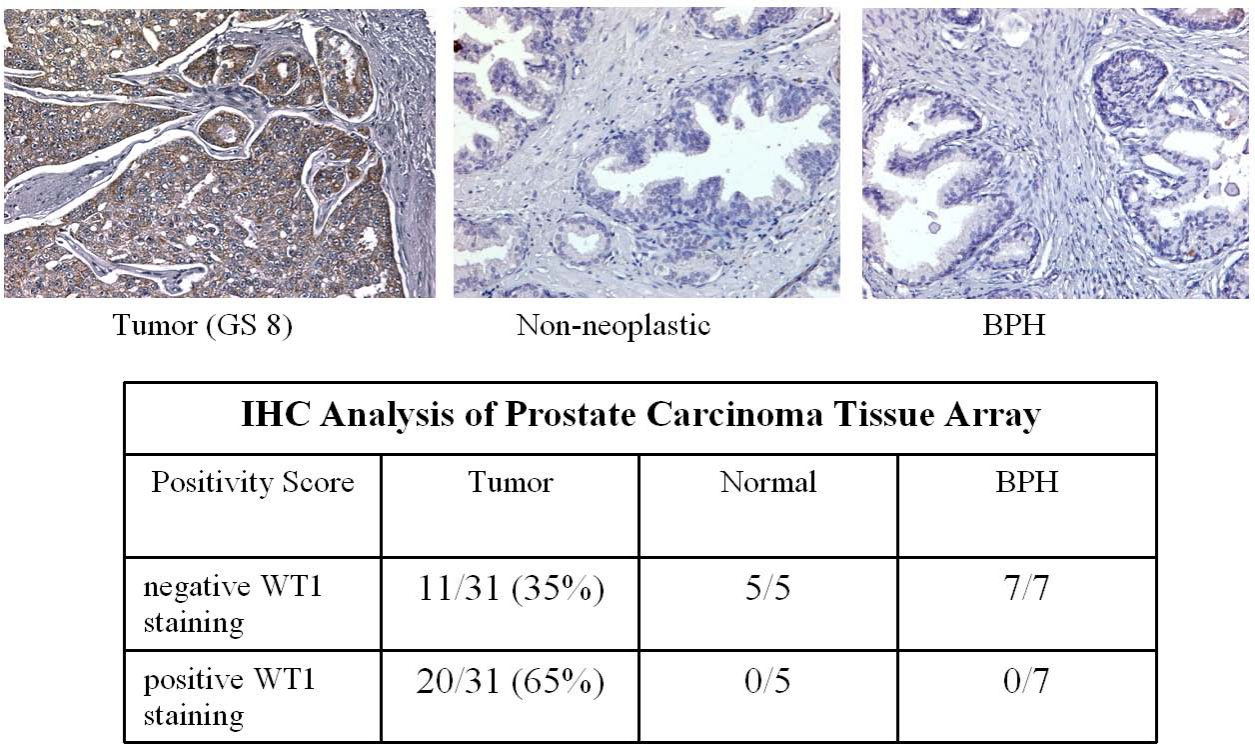
**Immunohistochemical analysis of WT1 expression in prostate tissue microarrays**. Top **Panel**. FFPE tissues were stained as described in text using WT1 polyclonal antibody (Epitomics). Representative fields of tumor tissue (left, Gleason Score 8) and normal (center) and BPH samples (right) panels show WT1 protein expression (brown) limited to tumor epithelium. **Bottom Panel**. Relative staining intensity was scored as described in text. Duplicated cores samples from 31 patients with Gleason score 6-10 (nearly half were high grade, Gleason 8-10) and 7 patients with BPH and 5 samples of normal tissue from cancer-free prostates were analyzed. Positive WT1 staining was seen in 65% of patient samples examined.

Because elevated levels of *WT1 *mRNA expression were observed in laser capture samples, frozen tissues, and tumorigenic cells, we examined WT1 protein expression by immunohistochemical analyses of prostate tissue microarrays. These results demonstrated the presence of WT1 protein in 65% of tumor samples examined (Figure 4). In contrast, WT1 protein was not detected in both normal prostate and benign prostatic hyperplasia samples (Figure 4 Top). Notably, in those samples with WT1 expression, the majority of staining was cytoplasmic with only a few samples demonstrating nuclear expression (not shown). Both cytoplasmic and nuclear WT1 staining has been shown in other tumor types [29,30].

## Discussion

Using laser capture microdissection to isolate distinct cell-type populations from epithelial and stromal tissues in prostate cancer, our results identified nearly 500 genes whose expression was significantly different between epithelial and stromal cells. One important finding was the differential expression of *WT1 *in prostate cancer epithelia cells. This cell specific expression suggests a potential role for *WT1 *in prostate cancer. While there have been reports of *WT1 *expression in prostate [29,31], our results demonstrate the most complete evidence of elevated *WT1 *expression at both mRNA and protein levels in prostate tumors. While Devilard et al. [32] demonstrated differential expression of *WT1 *by microarray analysis of the LuCaP cell line in a xenograft model, our study is the first to identify *WT1 *expression in microdissected human epithelial cells. We have confirmed the microarray results by real-time PCR and quantified *WT1 *expression in paired tissue samples and in established tumorigenic cell lines. In paired tumor and non-neoplastic tissue, *WT1 *expression was elevated in 70% of high-grade tumors examined. In three of four established prostate cancer cell lines, *WT1 *expression was also significantly higher than the non-neoplastic cell line RWPE-1. Further analysis of WT1 protein identified expression in 65% of tumor samples and, more importantly, the absence of expression in non-neoplastic and BPH samples.

This elevated *WT1 *expression provides evidence for a potential oncogenic role in prostate cancer. Although *WT1 *is expressed mainly in the urogenital system during development and in the central nervous system, bone marrow, lymph nodes, and gonads in adulthood [33,34], many studies have shown elevated *WT1 *expression in diverse cancer types [29], including leukemia [35-37]., breast [29,38,39], ovarian [40], mesothelioma and pulmonary adenocarcinomas [30]. Additionally, *WT1 *is being thoroughly investigated as a potential prognostic marker [35,38,41]. Structurally, WT1 belongs to the family of transcription factors with four Krüppel-like zinc fingers in the C-terminus that aid in nucleic acid binding. WT1 exists in multiple isoforms and its ability to regulate transcription is primarily determined by the presence or absence of three amino acids: lysine, threonine, and serine (KTS), encoded at the end of exon 9 [42]. Functionally, WT1 has been shown to regulate genes important in prostate cancer including VEGF, Bcl2, AR, and IGF1R [43-46]. We have recently identified potential WT1 binding sites in the regulatory sequences of genes expressed in prostate cancer epithelial cells [47,48]. Additionally, WT1 protein was identified bound to several of these gene promoters in native chromatin of transfected LNCaP cells. Therefore, an up-regulation of *WT1 *expression in prostate epithelial cells would be consistent with transcriptional modulation of important prostate cancer growth control genes.

In addition to nuclear WT1 protein, we and others have observed WT1 protein in the cytoplasm of several tumor types [30], and this is consistent with the presence of a cytoplasmic localization signal on the WT1 protein. Although the exact function of cytoplasmic WT1 remains to be elucidated, WT1 can shuttle between the nucleus and cytoplasm as it contains both a nuclear localization signal and a nuclear export signal [49]. One caveat is that cytoplasmic WT1 protein could be of one specific isoform, as antibody staining cannot distinguish amongst the various isoforms of the WT1 protein. It is possible that cytoplasmic protein is transcriptionally inactive, indeed the phosphorylated form is thought to be retained in the cytoplasm [50,51]. Another possibility is that the cytoplasmic function is post-transcriptional; surprisingly, it has been shown that both +KTS and -KTS isoforms can function as shuttling proteins and both associate with polyA RNPs and polysomes[52].

One surprising result was the pattern of *EGR1 *expression. Although *EGR1 *has previously been reported to be elevated in high grade prostate tumors (GS 8-10) [53], our results demonstrated that *EGR1 *expression was not significantly elevated in tumor tissues relative to non-neoplastic tissues in paired T3 stage samples. This trend was also consistent in cell cultures; the non-tumorigenic RWPE-1 cell line expressed greater levels of *EGR1 *than all tumorigenic cell lines tested. These discrepancies in *EGR1 *expression can primarily be attributed to two reasons. First, we measured *EGR1 *levels in paired samples within the same individual, while the aforementioned study examined tissue samples from unrelated individuals. Secondly, the tumor samples were all Gleason Score 7; so the possibility remains that *EGR1 *levels might be elevated in higher grade tumor samples. Clearly, the topic of *EGR1*'s activity as a tumor suppressor or oncogene remains highly debated [54].

Previous microarray studies have primarily examined prostate tumor tissues as a whole, containing both epithelial and stromal cell types, and compared their expression patterns to adjacent non-neoplastic tissue or normal donor prostates [9,10,55]. However, a comparison with the genes expressed significantly higher in our microdissected tumor epithelial samples suggests that some of the reported tumor genes in the literature are actually expressed in the stromal cell compartment and not in the epithelia. For example, SPARC expression appears in several tumor microarray analyses [56,57], but was identified in the stromal compartment in our studies and in other tumor types [58,59].

Our analysis of differential expression between adjacent stroma and tumor epithelia showed that the cytokines, *CCL5 *and *CXCL 13*, and the growth factors, *IGF-1 *and *FGF-2*, were upregulated in stromal cells. Additionally their expression was elevated in non-neoplastic paired frozen prostate tissues. Both IGF and FGF axes are known to be upregulated in prostate tumors [25-27] and several groups have shown *IGF-1 *to be expressed in prostate tumor stroma [26,60,61]. Overall our results are in agreement with other studies that have shown elevated expression of genes such *as IGF-1, FGF-2, IGFBP3, desmin, vinculin*, and *vimentin *in prostate stromal tissues [7,27,62]. These results demonstrate that genes differentially expressed in tumor cell compartments include those important to growth regulation, and in particular, genes of the IGF axis are expressed.

While it is difficult to make direct comparisons between this study and others that used LCM to examine altered expression in tumor *vs*. normal epithelia, we and others observed genes elevated in prostate cancer epithelial cells including *kallikrein proteins 2 (KLK2)*, and *3 (KLK3*, or *PSA) *[16]. *KLK2 *and *PSA *are androgen regulated serine proteases expressed in prostate epithelial cells and upregulated in prostate cancer [63]. Two ets related transcription factors observed in this study, *ets-related gene *(*ERG*) and *Sam pointed domain ets transcription factor (SPEDF*) [16] are known to be upregulated in prostate tumor epithelial cells [64,17,18]. The importance of the *ERG *gene is supported by its frequent involvement in complex rearrangements with a host of other gene fusion partners. Overall the expression of these genes in prostate cancer epithelial cells is consistent with their potential roles in tumorigenesis.

Fewer studies have used LCM to examine gene expression in stromal samples, but the SELECT cancer prevention trial identified expression of two angiogenesis genes elevated in stromal tissue: *angiopoietin1 (angpt1) *and the *endothelin A receptor (EDNRA)*, genes that we also observed in stromal tissues [14]. Additionally, gene families upregulated in normal stroma relative to reactive tumor stroma included: *caveolin (CAV), tropomyosin (TPM), transforming growth factor-B (TGFβ), Laminin (LAM)*, and *EDNR *[12]. In our study, *TPM1, TPM2, CAV1 *and *CAV2 *were elevated in stromal compared to epithelial tissue. Thus, while a direct comparison cannot be made between our unique study of tumor epithelial and stromal tissues and other studies focused predominantly on one tissue type, there are indications of common patterns of gene expression. Importantly, using this tissue specific approach novel gene expression patterns can be more clearly identified.

## Conclusions

In the present study, LCM and microarray analysis were used as tools to identify distinct gene expression patterns in prostate cell populations and led to the identification of genes of potential significance in prostate cancer, such as *WT1*. As *WT1 *has already been investigated as a clinical marker in acute leukemia, data demonstrating *WT1 *expression in prostate tumor tissues may point to its usefulness as a potential marker for prostate cancer.

## Data Deposition

Results of the microarray analyses are posted at NCBI's Gene Expression Omnibus and are accessible through GEO Series accession number GSE 20758 http://www.ncbi.nlm.nih.gov/geo/query/acc.cgi?acc=GSE20758.

## Abbreviations

The following abbreviations are used: *WT1: Wilms' Tumor 1; EGR1: early growth response 1; GATA2: GATA binding protein 2; CCL5: chemokine (C-C motif) ligand 5; CXCL13: chemokine (C-X-C motif) ligand 13; IGF-1: insulin-like growth factor 1; IFG-1R: insulin-like growth factor-1 receptor; IGFBP3: insulin-like growth factor binding protein 3; FGF-2: fibroblast growth factor-2; FGFR-3: fibroblast growth factor receptor-3*; LCM: laser capture microdissection; QRT-PCR: quantitative real time - polymerase chain reaction; IHC: immunohistochemistry; BPH: benign prostatic hyperplasia; OCT: optimal cutting temperature; FFPE: formalin-fixed paraffin embedded; TMA: tissue microarray; GO: gene ontology

## Competing interests

The authors declare that they have no competing interests.

## Authors' contributions

JLG participated in study design, performed the RNA and protein expression assays and drafted the manuscript, KEB participated in study design and performed the LCM and microarray assays, EMM contributed to the study design, performed the statistical analyses and helped to draft the manuscript, HP contributed to the study design, performed the bioinformatics analyses and helped to draft the manuscript. GCF conceived of the study, guided student research, participated in data analysis and drafted the manuscript. All authors read and approved the final manuscript.

## Pre-publication history

The pre-publication history for this paper can be accessed here:

http://www.biomedcentral.com/1471-2407/10/165/prepub

## Supplementary Material

Additional file 1 **List of genes significantly overexpressed in epithelial tissues as compared to stromal tissues**. This table lists 194 genes whose expression is significantly greater in epithelial cells than in stromal cells. Significance was measured by Bayesian paired t-test (using Cyber-T software) and this table is an extended version of Table 2 which ended at p < 10^-5^. Click here for file

Additional file 2 **List of genes significantly overexpressed in stromal tissues as compared to epithelial tissues**. This table lists 302 genes whose expression is significantly greater in stromal cells than in epithelial cells. Significance was measured by Bayesian paired t-test (using Cyber-T software) and this table is an extended version of Table 3 which ended at p < 10^-5^. Click here for file

Additional file 3 **Lists of Gene Ontology Biological Process (GO_BP) terms that are overrepresented in stromal (A) and epithelial (B) tissues (per DAVID analysis)**. This table lists gene functional classification clustering analysis of 194 genes that were elevated in epithelial cells and 302 genes elevated in stromal tissue (from Files 1 and 2, above). Clustering analysis was performed using DAVID and this table is an extension of Table 4 which listed only categories with enrichment scores >1.0. Click here for file
